# Short‐Term Surgical Outcomes: Modified Dorsal Inlay Graft Versus Tubularized Incised Plate Urethroplasty in Pediatric Hypospadias With Narrow Urethral Plates

**DOI:** 10.1111/andr.70166

**Published:** 2025-12-31

**Authors:** Yakun Xu, Yanmin Wan, Dianyong Liu, Bin Zhang

**Affiliations:** ^1^ Department of Pediatric Urology Dalian Women and Children's Medical Center (Group) Dalian China; ^2^ Department of Pediatric Urology Children's Hospital of Fudan University Shanghai China

**Keywords:** hypospadias, inlay graft, narrow urethral plate, reconstructive surgery, tubularized incised plate (TIP)

## Abstract

**Background:**

Reconstructing the urethra presents in hypospadias surgery is difficult. Occasionally, the urethral plate (UP) may be quite narrow, necessitating augmentation or replacement to achieve additional tubularization.

**Objective:**

The objective of this research was to assess the application of modified dorsal inlay graft (mDIG) and tubularized incised plate (TIP) urethroplasties in patients with hypospadias and narrow UPs and to determine which method yields superior functional and surgical outcomes.

**Materials and Methods:**

The medical records of 114 patients with distal/midshaft hypospadias and narrow UPs who were treated at our hospital from December 2020 to June 2024 were analyzed in this retrospective cohort study. These individuals were categorized into two distinct groups: the TIP and the mDIG groups. Standard preoperative characteristics, surgical technique, postoperative complication rate, maximum urinary flow rate (*Q*_max_), and the hypospadias objective scoring evaluation (HOSE) score were analyzed to assess treatment efficacy in both groups.

**Results:**

No significant differences in patient age, meatus location, UP width, coronal diameter, or urethral length were observed between the two groups. Sixty‐five patients (57.0%) underwent TIP urethroplasty, and 49 patients (43.0%) underwent mDIG urethroplasty. A statistically significant difference in operative time was observed, with operative times of 108 (101–118.5) and 85 (78–92.5) min (*p* < 0.001) in the mDIG and TIP groups, respectively. The mDIG group demonstrated a better HOSE score than the TIP group, with 5 (10.2%) versus 18 (27.7%) patients scoring less than 16 points and 44 (89.8%) patients versus 47 (72.3%) scoring 16 points, respectively. Three months after surgery, the *Q*_max_ was 11.7 ± 3.4 mL/s in the mDIG group versus 9.43 ± 3.1 mL/s in the TIP group (*p* < 0.05).

**Discussion and Conclusion:**

mDIG urethroplasty has a high success rate, ensuring both optimal aesthetic and functional outcomes in the short term, which, justifies the additional operative time.

## 1 Introduction

Hypospadias represents one of the most prevalent congenital anomalies affecting the male genitalia in pediatric populations. This condition is often linked with additional abnormalities in the ventral side of the prepuce and differing levels of curvature in the ventral penis. The primary approach to managing hypospadias is surgical intervention, which aims to correct the anatomical defects and restore normal function. However, despite the effectiveness of surgical treatment, patients may experience a range of complications that necessitate further surgical procedures. These complications can include fistula formation, diverticulum development, glans dehiscence, meatal stenosis, urethral stricture, and residual chordee. Fortunately, ongoing advancements in surgical techniques have significantly contributed to minimizing the incidence of these complications [1]. The complexity of hypospadias repair is compounded by challenges such as a narrow urethral plate (UP) and a flat glans, which prevent straightforward tubularization. To address this, the technique known as tubularized incised plate (TIP) urethroplasty, commonly used for repairing distal hypospadias, demonstrates positive results. However, it is linked to a considerable occurrence of meatal stenosis and neo‐urethral strictures, especially in instances where the UP is narrow [2]. The width of the UP has been identified as a crucial risk factor contributing to complications associated with urethroplasty after performing TIP urethroplasty for hypospadias. Holland and Smith and Sarhan et al. [3, 4] reported that a pre‐incision UP width of less than 8 mm significantly increased the likelihood of urethroplasty complications after TIP repair.

In 2000, Kolon and Gonzales Jr. utilized dorsal inlay grafts (DIGs) in incised areas of narrow UPs during hypospadias repair to reduce the incidence of meatal stenosis and neo‐urethral stricture [5]. A narrow UP and shallow glans are key indications for graft use. However, the application of a longer free graft requires advanced surgical skills and carries a greater risk of graft necrosis [6].

Consequently, we improved the initial surgical method and carried out this research to assess the effectiveness and results of modified dorsal inlay graft (mDIG) urethroplasty compared with TIP in individuals with narrow UPs, to determine which repair technique yields superior results.

## 2 Materials and Methods

We performed a retrospective examination of data involving 114 consecutive pediatric patients who underwent mDIG and TIP procedures carried out by the same surgeon (Bin Zhang) at the Urology Department of the Children's Hospital of Fudan University (Shanghai, China) from December 2020 to June 2024. Patients with recurrent issues, circumcision, wide UPs (≥ 6 mm) [7], a history of testosterone therapy for small glans or other reasons, and a penile chordee greater than 30° were excluded. The inclusion criteria were as follows: (1) male patients with primary distal/midshaft hypospadias confirmed by two senior pediatric urologists; (2) a UP width < 6 mm as measured by a Vernier caliper under artificial erection; (3) a ventral penile curvature ≤ 30° after degloving; (4) no history of testosterone therapy or circumcision; (5) complete clinical data and follow‐up ≥ 4 months; and (6) written informed consent provided by guardians. This research adhered to the Declaration of Helsinki (revised in 2013) and received approval from the Ethics Board of the Children's Hospital of Fudan University {No. [2021]531}, with informed consent obtained from the parents of all participants. To evaluate treatment efficacy in both groups, we analyzed several parameters: standard preoperative characteristics, the surgical technique employed, and the postoperative complication rate. Functional and aesthetic outcomes were assessed using the hypospadias objective scoring evaluation (HOSE) at 2 months, which incorporates both traditional functional criteria—such as a single urinary stream, straight erection, and absence of fistula—and contemporary aesthetic considerations, including a vertically oriented meatus situated close to the tip of the glans [8]. For statistical analysis, complications were categorized based on the basis of their presence at the time of HOSE assessment (2 months), while delayed resolution was noted separately. In addition, the maximum urinary flow rate (*Q*_max_), a key urodynamic measure defined as the highest instantaneous speed of urine expulsion during a complete voiding cycle, was evaluated [9]. A reduced *Q*_max_ often suggests possible urinary tract obstruction.

### 2.1 Operative Techniques

**FIGURE 1:**
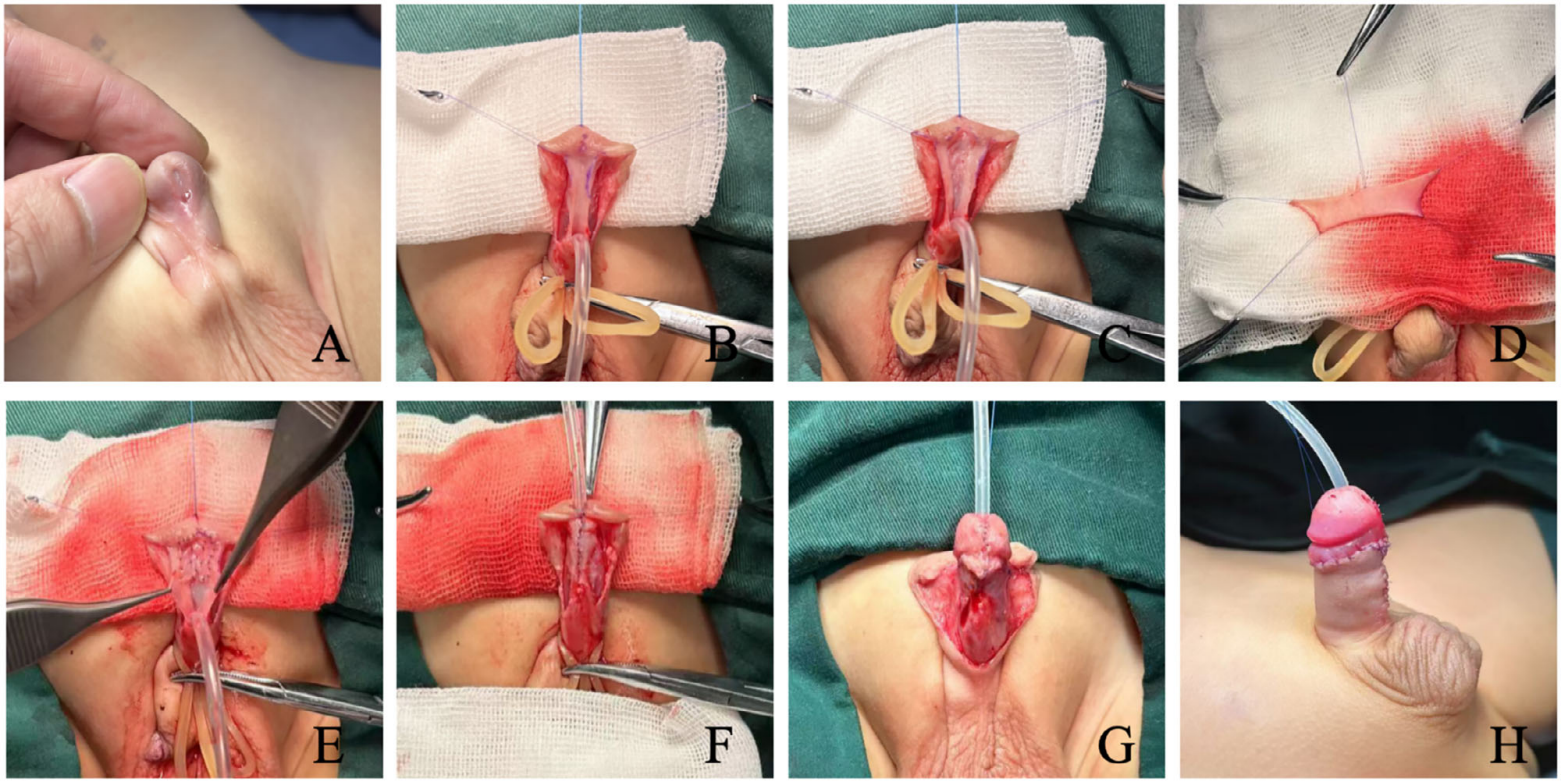
Intraoperative photos (mDIG): (A) Preoperative right oblique view. (B) Dissecting the divergent corpus spongiosum with Buck's fascia at each side to the 3–9 o'clock position. (C) Incising the urethral plate. (D) Prepuce inner plate skin graft. (E) Inlay grafting, the incision of the proximal UP wound is left undisturbed. (F) Tubularized urethroplasty. (G) Spongioplasty with Buck's fascia. (H) Postoperative appearance.

All interventions were carried out by separate pediatric urologists. Before surgery, intravenous antibiotic prophylaxis (cefuroxime at a dosage of 30 mg/kg IV) was given. To manage postoperative discomfort, a caudal block was applied. The patients were positioned supinely with a right oblique alignment before the procedure (Figure 1A). A vertical holding suture was applied to the glans, which was subsequently infiltrated with epinephrine at a concentration of 1:100,000 along the edges of the UP. The incision created here facilitated the formation of ventral neo‐urethral flaps and the elevation of the glans wings. The membranous urethra was resected at the urethral meatus. A superficial U‐shaped incision was made around the meatus, accompanied by parallel incisions along both sides of the UP, extending to the mucosal collar. Following this, a circular incision was created around the corona. The penile skin was removed, and parallel incisions were made down to the corpora cavernosa on either side of the UP, extending from the tip of the glans to where the corpus spongiosum divides proximally. Sections of the corpus spongiosum were carefully dissected with Buck's fascia on both sides, reaching outward to the 3–9 o'clock positions. When the corpus spongiosum contributed to the ventral curvature, it was transected (Figure 1B). After ventral penile release was completed, a test for artificial erection was conducted. Persistent ventral curvature was corrected by plicating the tunica albuginea at the 12 o'clock position on the dorsal aspect, exactly at the area of greatest curvature.

### 2.2 TIP Group

TIP urethroplasty, as described by Snodgrass [10], was performed. In collaboration with colleagues, the procedure began with a careful incision along the midline of the UP, stretching from the meatus to the apex and extending deeply into the corpora cavernosa. To reconstruct the urethra, it was performed in two subepithelial layers using 7/0 polydioxanone with continuous sutures.

### 2.3 mDIG Group

A midline dorsal incision was made, as in TIP urethroplasty, and the defect was grafted (Figure 1C). The graft, taken from the inner prepuce (Figure 1D), had dimensions of 6–8 mm in width and 8–10 mm in length, and it was placed into the wound at the distal glans. The incision of the proximal UP wound was left undisturbed, allowing it to epithelialize and heal naturally (Figure 1E). The graft was defatted and secured to the perimeter of the incision using 7/0 polydioxanone. Extra quilting stitches using 7/0 polydioxanone secure the graft to the underlying corpora cavernosa at the midline and on both sides, spaced roughly 2 mm apart. Two‐layer tubularization was performed using subepithelial 7/0 polydioxanone sutures (Figure 1F).

The procedures of spongioplasty and glanuloplasty involved stitching the two edges of the corpus spongiosum to the midline, while the neourethra was secured using interrupted subcuticular sutures made of 6/0 polyglactin (Figure 1G,H). For urinary drainage, a silicone Foley catheter ranging from 6 to 10 Fr was employed in every patient, and the surgical area was covered with gauze bandages. For all the children, the sterile dressing was removed on postoperative Day 5, and the urinary catheter was removed on postoperative Day 14.

All postoperative patients were followed up regularly at 1 month, 2 months, 3 months, 6 months, and annually thereafter. Follow‐up data were collected through a combination of outpatient visits and social media platforms. During these sessions, photographs of the affected area and video recordings of urination were obtained. Surgical outcome evaluation was done according to the HOSE score at the second month postoperatively. Additionally, children older than 3 years underwent urinary flow rate assessments 3 months postoperatively.

### 2.4 Statistical Analysis

First, to assess the distribution of the data, the Shapiro–Wilk test, which is a standard method for determining whether the data follow a normal distribution, was employed. Second, means with standard deviations, medians and IQRs or frequencies and proportions were calculated for continuous normally distributed variables, continuous non‐normally distributed variables, or categorical variables, respectively. Third, when comparing continuous variables, the analysis utilized either the unpaired *t*‐test or the Mann–Whitney *U*‐test, which are appropriate for assessing differences between two independent groups. For categorical variables, the analysis was conducted using chi‐square tests or, when necessary, two‐tailed Fisher's exact tests. A *p* value of < 0.05 was considered to indicate statistically significant. Data analysis for this study was conducted utilizing IBM SPSS Statistics software, version 30.0.

## 3 Results

### 3.1 Descriptive Characteristics

This study involved a total of 114 patients who were treated consecutively. The participants were categorized into two distinct groups: the mDIG group, which included 49 individuals (43.0%) who underwent DIG urethroplasty, and the TIP group, consisting of 65 individuals (57.0%) who underwent TIP urethroplasty. Both groups were similar in terms of age, UP width, coronal diameter, and the location of the meatus, as detailed in Table 1 (*p* > 0.05). There was no significant difference in UP width between the two groups (*p* = 0.093), with all individuals exhibiting a UP width of under 6 mm.

**TABLE 1:** Patient demographics and outcomes in the study groups.

Characteristic	mDIG	TIP	*p*
**Patients**	49 (43%)	65 (57%)	
**Age (months)**	24 (13–46.5)	23 (13–35)	0.472
**Width of urethral plate (mm)**	4 (4–5)	5 (4–5)	0.093
**Diameter of coronal incision (mm)**	10 (10–10)	10 (10–11)	0.303
**Meatus location**			0.994
Distal shaft	3 (6.1%)	4 (6.2%)	
Midshaft	46 (93.9%)	61 (93.8%)	
**Operative time (min)**	108 (101–118.5)	85 (78–92.5)	< 0.001^a^
**Dorsal plication**	2 (4.1%)	3 (4.6%)	0.890
**Length of urethral (cm)**	1.8 (1.5–2.0)	1.6 (1.5–2.0)	0.350
**Success**	45 (91.8%)	57 (87.7%)	
**Overall complications**	4 (8.2%)	8 (12.3%)	0.641
Stenosis	—	1 (1.5%)	
Fistula	4 (8.2%)	6 (9.3%)	
Glans dehiscence	—	1 (1.5%)	
Diverticulum	—	—	

Data are presented as medians and interquartile ranges (IQRs) or *n* (%).

Abbreviations: mDIG: modified dorsal inlay graft; TIP, tubularized incised plate.

^a^ Significant.

### 3.2 Surgical Outcomes

The only notable statistical difference identified between the groups was in the duration of the surgery, which was greater for the mDIG group (108 [101–118.5] min) compared with the TIP group (85 [78–92.5] min; *p* < 0.001), as illustrated in Table 1. No significant differences were found between the groups in terms of dorsal plication or urethral length. Overall, complications were reported in 12 (10.5%) children. The only complication observed in the mDIG group was fistula formation in four (8.2%) patients, whereas success was documented in 45/49 (91.8%) patients. Within the TIP group, a successful outcome was noted in 57 out of 65 patients, representing 87.7% (*p* > 0.05). Fistulas were identified in six individuals (9.3%), while one individual (1.5%) developed urethral stenosis and another (1.5%) experienced ventral dehiscence of the neourethra. Neither group exhibited any instances of neo‐urethral stenosis or diverticulum. One patient in the mDIG group had a urethral fistula that healed spontaneously 3 months postoperatively. The patients underwent HOSE score 2 months after surgery, and all patients were included in the analysis regardless of subsequent complication resolution (e.g., spontaneous fistula healing at 3 months). The results indicated that the distal glandular position was more prevalent in the mDIG group (100%) than in the TIP group (83.1%), with this difference being statistically significant. Additionally, the mDIG group had an overall HOSE score that was notably higher than that of the TIP group (*p* = 0.021; Table 2). Table 3 presents the evaluation of the *Q*_max_ at the third postoperative month. Among the 114 patients, 46 (40.4%) had their *Q*_max_ assessed during follow‐up, while the remaining individuals had not achieved toilet training by their final postoperative appointment. The average *Q*_max_ recorded was 11.7 ± 3.4 mL/s for the mDIG group, compared with 9.43 ± 3.1 mL/s for the TIP group, revealing a statistically significant difference between these two groups (*p* = 0.020).

**TABLE 2:** Postoperative cosmetic assessment (hypospadias objective scoring evaluation score) at second month.

	mDIG	TIP	*p*
**Meatal location**			0.01^a^
Distal glans	49 (100%)	54 (83.1%)	
Proximal glans	0	10 (15.4%)	
Coronal	0	1 (1.5%)	
**Meatal shape**			0.383
Vertical slit	49 (100%)	63 (96.9%)	
Circular	0	2 (3.1%)	
**Urinary stream**			0.288
Single steam	48 (98.0%)	61 (93.8%)	
Spray	1 (2.0%)	4 (6.2%)	
**Erection**			—
Straight	49 (100%)	65 (100%)	
**Fistula**			0.943
None	45 (91.8%)	59 (90.8%)	
Single‐subcoronal or more distal	3 (6.1%)	4 (6.2%)	
Single‐proximal	1^b^ (2.1%)	2 (3.0%)	
**Total score**			0.021^a^
< 16	5 (10.2%)	18 (27.7%)	
16	44 (89.8%)	47 (72.3%)	

The data are presented as *n* (%).

Abbreviations: mDIG, modified dorsal inlay graft; TIP, tubularized incised plate.

^a^ Significant.

^b^ Healed by itself at third month.

**TABLE 3:** Postoperative maximum urinary flow rate (*Q*_max_) at 3 months (age ≥ 36 months).

	mDIG	TIP	*p*
**Patients**	21 (42.9%)	25 (38.5%)	
***Q*_max_ (mL/s)**	11.7 ± 3.4	9.43 ± 3.1	0.020^a^

The data are presented as the means ± standard deviations or *n* (%).

Abbreviations: mDIG, modified dorsal inlay graft; TIP, tubularized incised plate.

^a^ Significant.

## 4 Discussion

Snodgrass first presented the TIP technique for distal hypospadias in situations where the urethral groove lacked sufficient depth for straightforward tubularization [10, 11]. This technique has since gained widespread popularity, with large series being published as experience has increased, demonstrating varying outcomes. When utilized in a wide range of distal hypospadias cases, the TIP technique has demonstrated positive cosmetic outcomes, with complication rates reported as low as 0% for stenosis, 2% for fistulas, and 2% for glans dehiscence among a cohort of more than 400 patients [12]. Holland and Smith [3] initially indicated that a UP width of under 8 mm after a pre‐relaxing incision correlated with an increased likelihood of fistula formation in individuals who underwent distal TIP urethroplasty, with this issue noted in all patients exhibiting such plate measurements. Later, Sarhan and his team [4] also determined that although various UP features had a statistically insignificant impact on the overall complication rate, having an adequate UP width of 8 mm or more was essential for the success of TIP repair. Nonetheless, penile dimensions differ among ethnic groups, with Chinese patients generally exhibiting slightly smaller penises than their Western counterparts [13]. According to our preliminary research, employing this technique for patients with a UP width ≥ 6 mm has been shown to be sufficient to achieve successful hypospadias repair outcomes [7]. Conversely, a narrow UP heightens complication risk, including meatal or neo‐urethral stenosis, as previously reported in several studies [14]. Therefore, in this study, urethral stricture is defined as a urethral diameter < 6 mm.

Kolon and Gonzales Jr. [5] were the first to suggest the use of grafts on incised UPs to enhance healing via epithelialization. Drawing from their work with 32 patients, they found that grafting helps to avert complications such as meatal stenosis, neo‐urethral stricture, fistula, and urethral diverticulum, particularly when a narrow UP is present. The DIG technique is an advancement of the traditional TIP method, aimed at facilitating the healing of the incised urethra. During TIP urethroplasty, the healing of the dorsal UP incision occurs through the process of re‐epithelialization, which involves the growth of normal tissue, while closures made with sutures tend to heal with a desmoplastic and inflammatory response [15]. It has been suggested that the presence of a large, exposed area within the neourethra, which undergoes re‐epithelialization and scarring, could significantly contribute to the occurrence of meatal or neo‐urethral stenosis. The introduction of a free inlay graft is intended to maintain the integrity of the UP and expand the region of healthy tissue. Owing to its complexity, DIG urethroplasty is more technically demanding than the conventional TIP urethroplasty technique, requiring appropriately tailored indications. Measuring the urethral ratio before and after UP incision helps determine the necessity of grafting; if the ratio is less than 0.5, a graft should be applied, as the raw area would constitute a larger portion of the urethral circumference, increasing the risk of stricture [16]. Longer free grafts necessitate advanced surgical skills and carry the risk of graft necrosis [6]. Additionally, this method also results in a cavity‐occupying effect, leading to a reduction in the cross‐sectional area of the urethral cavity. We have concentrated on discovering strategies to alleviate the technical difficulties associated with DIG urethroplasty while also aiming to lower the rates of complications. In this context, we view patients with less favorable anatomical features, such as narrow and poor spongiosum, as the most suitable candidates. To enhance the DIG procedure, we selectively embedded only the 8–10 mm wound at the anterior aspect of the penile incision with a free preputial skin graft. This approach ensures complete epithelialization of the penile skin incision, effectively preventing urethral orifice narrowing due to ongoing wound healing. In contrast, the wound at the proximal UP incision was left fully open to allow for natural healing processes.

In this study, we compared two procedures for hypospadias in patients with primary distal hypospadias and narrow UPs, dividing the patients into two groups. The mDIG group comprised 49 individuals who received mDIG repair, while the TIP group had 65 individuals who underwent ventral TIP repair. Both were similar regarding patient age, meatus position, UP depth, coronal diameter, dorsal plication, and urethral length.

The operative time was 108 (101–118.5) min in the mDIG group and 85 (78–92.5) min in the TIP group, indicating that the operative time of mDIG urethroplasty is significantly longer than that of conventional TIP urethroplasty.

In alignment with our research findings, both Helmy et al. [17] and Mouravas et al. [18] have documented an increase in surgical duration for patients undergoing the DIG procedure. Notably, our operative times are superior to those reported in previous studies.

Few studies have examined the outcomes of TIP urethroplasty in patients with narrow UPs. Eldeeb et al. [19] reported that of 30 patients with narrow UPs who underwent TIP urethroplasty, one developed meatal stenosis, while another developed a distal penile fistula. Conversely, Sarhan et al. [4] found higher complication rates in patients with UP widths under 8 mm out of 80 cases. Additionally, Ali et al. [20] found an 87.5% success rate within the inlay graft urethroplasty group (*n* = 40), with glandular dehiscence observed in one patient (2.5%), fistulas in two patients (5%), and a narrow meatus in another two patients (5%). Moreover, the TIP group (*n* = 40) exhibited a lower success rate of 62.5%, with glandular dehiscence occurring in eight patients (20%), fistulas occurring in five patients (12.5%), and narrow meatus occurring in seven patients (17.5%), including five patients who presented with both narrow meatus and fistula.

In our research, the total complication rate for the mDIG group (*n* = 49) was recorded at 8.5%, with fistulas developing in four individuals. Conversely, the TIP group (*n* = 65) exhibited a fistula incidence of 9.3% (*n* = 6), along with one case each of glans dehiscence and urethral stenosis, both at 1.5%. There were no diverticulum occurrences. One case of urinary retention secondary to urethral stenosis was effectively managed with indwelling stent placement. Notably, no persistent urethral meatus issues were observed. One patient in the mDIG group developed a urethral fistula that healed spontaneously by 3 months postoperatively. These results are encouraging. The relatively low incidence of complications in our study may be attributed to the use of Buck fascia reconstruction, which provides robust coverage and enhanced vascular supply to the newly formed urethra [21]. This technique has favorable short‐term outcomes, eliminating the need to rely on residual preputial blood supply and enabling simpler, safer, and more aesthetically pleasing penile coverage. Thus, mDIG urethroplasty represents a promising option for managing distal penile hypospadias in children with narrow UPs.

The evaluation of hypospadias repair should take into account both aesthetic and functional results. Holland et al. [22] developed HOSE that emphasizes traditional functional criteria—such as a single urinary stream, straight erection, and the absence of fistula—while also reflecting contemporary concerns regarding aesthetics, including positioning a vertically oriented meatus close to the glans tip. In this research, the functional and cosmetic results assessed through the HOSE score showed that four (8.3%) patients achieved scores below 16 points, while 44 (91.7%) patients reached 16 points in the mDIG group. In contrast, the TIP group had 18 (27.7%) patients scoring below 16 points and 47 (72.3%) patients scoring 16 points, suggesting that mDIG urethroplasty is linked to better overall objective outcomes.

Due to the customary approach of initiating toilet training early in Asian households [23], a majority of Chinese parents strive to finish this process within the initial 3 years of their child's life. All patients over the age of three diagnosed with hypospadias underwent a uroflowmetry assessment during the follow‐up phase after surgery. In the short‐term follow‐up, the average *Q*_max_ recorded was 11.7 ± 3.4 mL/s for the mDIG group, while the TIP group showed a mean of 9.43 ± 3.1 mL/s, with a statistically significant difference observed. These results are in close agreement with those presented by Kim et al. [24] and Andersson et al. [25], who indicated that urinary flow normalized in the short term when utilizing the Miskolc nomogram for patients treated with DIG. Nevertheless, the flow rate measurements taken at the 1‐year mark did not show any significant differences between the two groups concerning *Q*_max_ and voiding duration [17].

A key constraint of our research stems from its retrospective nature. Additional randomized controlled trials are essential to confirm that mDIG urethroplasty is a safe and effective treatment for children with narrow UPs undergoing revision hypospadias repair. Additionally, the outcomes, including complications, from this single‐surgeon series cannot be generalized, as they may be influenced by the surgeon's specific skill set. Moreover, long‐term follow‐up is essential to evaluate voiding function comprehensively. We intend to maintain our observation of these patients as they progress through adolescence and into adulthood. This ongoing monitoring will allow us to evaluate the long‐term effects on their overall health, particularly focusing on aspects related to ineffective functioning and sexual health. Recent studies, including a multicenter investigations on preputial spiral graft urethroplasty, have emphasized that harvesting preputial tissue at a young age could compromise its availability for future complex urethral reconstructions, such as in pan urethral stricture repair [26]. This important aspect warrants further consideration within the context of our research on preputial graft applications in very young patients.

## 5 Conclusions

Based on our research, mDIG urethroplasty proves to be a reliable and efficient technique for repairing distal penile hypospadias in children with narrow UPs. This approach may reduce complications often associated with the TIP technique in hypospadias repair. This technique has a high success rate, delivering both optimal aesthetic and functional outcomes in the short term, which, in our view, justifies the additional operative time.

## Author Contributions

Data curation: Yakun Xu, Yanmin Wan, Bin Zhang. Writing – original draft: Yakun Xu, Yanmin Wan. Writing – review and editing: Bin Zhang, Dianyong Liu. Project administration: Bin Zhang.

## Funding

This research was funded by the National Clinical Key Specialty Construction Project (grant no. 10000015Z155080000004). This trial was registered with ClinicalTrials.gov (NCT04533477).

## Consent

Written informed consent was obtained from the patient for publication of this case series and any accompanying images.

## Conflicts of Interest

The authors declare no conflicts of interest.

## Data Availability

The data are available from the corresponding author upon request.
